# Associação entre Estimativa de Colesterol de Lipoproteína de Baixa Densidade (sdLDL-C) e Risco de Doença Cardiovascular Aterosclerótica

**DOI:** 10.36660/abc.20240265

**Published:** 2025-01-22

**Authors:** Shutang Zhang, Jinjie Du, Peng Wang, Min Lei, Canye Zhong, Yang Ou, Zhen Sun

**Affiliations:** 1 Chongqing University Fuling Hospital Chongqing Clinical Research Center for Geriatric Diseases Chongqing China Chongqing University Fuling Hospital, Chongqing Clinical Research Center for Geriatric Diseases – Geriatrics, Chongqing – China

**Keywords:** Lipoproteínas, Aterosclerose, Fatores de Risco

## Abstract

**Fundamento:**

Uma nova fórmula para estimar o colesterol de lipoproteínas pequenas, densas e de baixa densidade (sdLDL-C) com base nos resultados do painel lipídico padrão é proposto.

**Objetivos:**

Para avaliar a associação entreestimado sdLDL-C (EsdLDL-C) e o risco de doença cardiovascular arterosclerótica (DCVA).

**Métodos:**

Um total de 12.192 participantes do banco de dados do *Korea National Health and Nutrition Examination Survey (KNHANES)* entre 2010 e 2020 foram incluídos neste estudo transversal. EsdLDL-C foi calculada como EsdLDL-C = LDL-C- [1,43 × LDL-C - (0,14 × (ln (TG) × LDL-C)) - 8,99]. Análises de regressão logística foram utilizadas para avaliar a associação entre EsdLDL-C e risco de DCVA. As análises de subgrupos foram realizadas com base na idade, índice de massa corporal (IMC), hipertensão, e diabetes. Uma razão de possibilidades (OR) com um intervalo de confiança de 95% (IC) foi utilizado para avaliação. P<0,05 foi considerado estatisticamente significativo.

**Resultados:**

Entre 12.192 participantes, 1.239 (10,16%) tinham DCVA. A média de sdLDL-C dos participantes foi estimada em 42,43±14,75 mg/dL usando a fórmula. Níveis elevados de EsdLDL-C (OR=1,33; IC 95%, 1,06-1,66) foram associados a um aumento do risco de DCVA. As análises de subgrupos descobriram que pode haver uma interação entre EsdLDL-C (P_interação_=0,001) ou não-HDL-C (P_interação_=0,015) e hipertensão no risco de DCVA.

**Conclusões:**

Níveis elevados estimados de sdLDL-C foram associados ao risco de DCVA, e o sdLDL-C estimado pode ser uma alternativa à medição do sdLDL-C para avaliação do risco de DCVA.

## Graphical abstract

**Figura Central:**
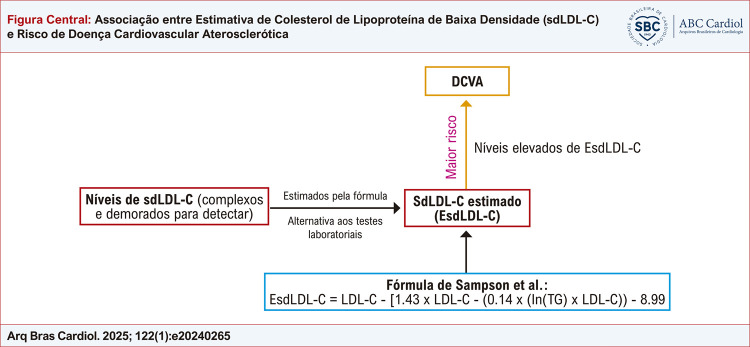
: Associação entre Estimativa de Colesterol de Lipoproteína de Baixa Densidade (sdLDL-C) e Risco de Doença Cardiovascular Aterosclerótica

## Introdução

A doença cardiovascular aterosclerótica (DCVA) é uma doença insidiosa e crônica que geralmente progride para um estágio avançado quando os sintomas aparecem.^1^ As doenças mais frequentes da DCVA são a doença cardíaca coronária e o acidente vascular cerebral, que são a principal causa de morte.^2^ A Organização Mundial da Saúde relatou que a DCVA se tornou a principal causa de morte em todo o mundo, ceifando aproximadamente 17,9 milhões de vidas a cada ano.^1^ A identificação e o monitoramento de biomarcadores associados à DCVA desempenham um papel importante na sua prevenção primária e secundária.

O aumento do colesterol de lipoproteína de baixa densidade (LDL-C) é um fator causal fundamental no desenvolvimento e progressão da DCVA.^3,4^ Estudos anteriores demonstraram que indivíduos com baixos níveis de LDL-C têm menor incidência de DCVA do que aqueles com altos níveis de LDL-C.^5-7^ O LDL é composto por várias subclasses de partículas com diferentes tamanhos e densidades, incluindo LDLs grandes flutuantes (lb) e intermediárias e pequenas densas (sd).^8^ No entanto, o sdLDL pode ser um biomarcador melhor do que outros subtipos para risco de DCVA em diferentes subtipos de LDL.^9,10^ Foi relatado que o sdLDL está associado a uma variedade de doenças, incluindo distúrbios metabólicos, obesidade e diabetes tipo 2, e é considerado um fator de risco para doença cardíaca coronária.^11-13^ Portanto, a medição dos níveis de sdLDL-C é de grande importância no monitoramento do risco de DCVA. Os métodos tradicionais de medição de sdLDL-C dependiam de ultracentrifugação complexa ou eletroforese em gel de gradiente.^14^ O equipamento especial necessário para medição e longo tempo de ensaio limitou a aplicação clínica da medição de sdLDL. Sampson et al. desenvolveram uma nova equação para estimar sdLDL-C com base nos resultados do painel lipídico padrão com um coeficiente de determinação de 0,745.^15^ No entanto, sua fórmula só foi estabelecida na população estadunidense, e a adaptação e o efeito estimado em outras populações permanece pouco claro.

Aqui, levantamos a hipótese de que a fórmula de Sampson et al. para estimar sdLDL também era aplicável a outras populações e estava associada ao risco de DCVA. Dados do banco de dados do *Korea National Health and Nutrition Examination Survey (KNHANES)* foram usados para avaliar a associação entre sdLDL e risco de DCVA.

## Métodos

### Aquisição de dados e participantes

Os dados utilizados no estudo transversal foram extraídos do banco de dados KNHANES entre 2010 e 2020.^16^ O banco de dados KNHANES é um sistema nacional de vigilância para avaliar a saúde e o estado nutricional dos coreanos, coletando informações sobre status socioeconômico, comportamentos relacionados à saúde, qualidade de vida, utilização de serviços de saúde, medidas antropométricas e perfis bioquímicos e clínicos de doenças não transmissíveis.^17^ KNHANES é uma pesquisa transversal nacionalmente representativa conduzida anualmente, cada ano de pesquisa incluindo uma nova amostra de aproximadamente 10.000 pessoas com 1 ano ou mais. A pesquisa consiste em três partes: entrevista de saúde, exame de saúde e pesquisa nutricional. A entrevista de saúde e o exame de saúde são conduzidos em um centro de exames móvel por equipe médica treinada e entrevistadores. Uma semana após o exame de saúde, nutricionistas foram às casas dos participantes para uma pesquisa nutricional. Participantes com mais de 18 anos e com informações completas sobre colesterol foram incluídos. Participantes foram excluídos com base nos seguintes critérios: (1) com valores anormais de IMC (IMC >40kg/m^2^); (2) com falta de informação sobre hemoglobina glicada (HbA1c); (3) com informações faltantes sobre acidente vascular cerebral ou doença cardíaca isquêmica. Os protocolos do KNHANES foram aprovados pelos Centros de Controle e Prevenção de Doenças da Coreia (KCDC). Todos os dados usados neste estudo são anonimizados no banco de dados do KNHANES e não envolveram intervenções humanas. Portanto, este estudo não exigiu aprovação adicional do Conselho de Revisão Institucional.

### Coleta de dados

Indicadores demográficos e bioquímicos dos participantes incluem idade (≥18 anos), gênero (masculino e feminino), índice de massa corporal (IMC), nível de renda [quartis (Q1, Q2, Q3, Q4) e desconhecido], nível de escolaridade (seodang/hanhak, sem educação, ensino fundamental, ensino médio e desconhecido), consumo de álcool (sim, não e desconhecido), tabagismo (sim e não), medicamento hipolipemiante (sim, não e desconhecido), hipertensão (sim, não e desconhecido), diabetes (sim, não e desconhecido), creatinina, nitrogênio ureico no sangue (BUN), HbA1c, LDL-C, lipoproteína de alta densidade (HDL-C), não-HDL-C, triglicerídeos (TG), colesterol total

(CT) e sdLDL-C estimado (EsdLDL-C) foram coletados. O não-HDL-C foi calculado como não-HDL-C = CT - HDL-C. Todos os níveis de lipídios foram medidos por coleta direta de sangue por uma enfermeira, no contexto de participantes que jantaram no dia anterior.

## Definição e medição

### DCVA

Os eventos de DCVA incluem infarto do miocárdio, angina, intervenção coronária percutânea, enxerto de bypass da artéria coronária, insuficiência cardíaca congestiva, doença vascular periférica, acidente vascular cerebral e ataque isquêmico transitório. Devido às limitações do banco de dados KNHANES, os eventos de DCVA incluíram doença cardíaca isquêmica, infarto do miocárdio, angina de peito e acidente vascular cerebral. No banco de dados KNHANES, a doença cardíaca isquêmica foi determinada pela pergunta: “Você já foi diagnosticado com infarto do miocárdio ou angina pelo seu médico?”. Portanto, os eventos de DCVA neste estudo incluíram apenas doença cardíaca isquêmica e ACV.

### sdLDL-C

sdLDL-C foi calculado a partir de Sampson et al.^15^. As fórmulas relevantes foram as seguintes:


$$\begin{array}{cc} & \text{ lbLDL-C }=1,43\times \text{ LDL-C }-(0,14\times (\ln(TG)\times \text{ LDL-C) })-\\ & 8,99 \end{array}$$ (1)

sdLDL-C = LDL-C - IbLDL-C (2)

### Análise estatística

Variáveis contínuas foram testadas quanto à normalidade usando o método de assimetria e curtose, e o teste de Levene foi usado para testar a homogeneidade da variância. Variáveis contínuas distribuídas normalmente foram descritas por média e desvio padrão (DP). A comparação entre grupos de variáveis contínuas com variâncias homogêneas foi realizada usando o teste t de Student não pareado, e variáveis contínuas com heterocedasticidade foram realizadas usando o teste t de Satterthwaite. Variáveis contínuas distribuídas não normalmente foram descritas por mediana e quartil [M (Q1, Q3)], e o teste de soma de postos de Wilcoxon foi usado para comparações intergrupos. Variáveis categóricas foram apresentadas por números e a razão constituinte [n (%)], e a comparação entre grupos foi realizada usando o teste qui-quadrado ou teste exato de Fisher.

Foi realizada uma análise de diferença entre as características dos participantes com e sem DCVA. Variáveis com p < 0,05 na análise de diferença foram rastreadas por regressão bidirecional por etapas, e as variáveis finais rastreadas foram ajustadas em análise de regressão logística multivariável. Análises de regressão logística univariável e multivariável foram usadas para avaliar a associação de EsdLDL-C, não-HDL-C, HDL-C, LDL-C, TG e CT com o risco de DCVA, acidente vascular cerebral e doença cardíaca isquêmica. As associações foram expressas como razão de chances (OR) com intervalo de confiança (IC) de 95%. A área sob a curva ROC (AUC) foi usada para avaliar a capacidade de EsdLDL-C, não-HDL-C, HDL-C, LDL-C, TG e CT de prever o risco de DCVA, e o teste DeLong foi usado para comparar as diferenças na AUC entre esses indicadores. A análise de subgrupos foi conduzida com base na idade (<65 e ≥65), IMC (<24,44 e ≥24,44 kg/m^2^), hipertensão (não e sim) e diabetes (não e sim). As análises estatísticas foram realizadas pelo software SAS 9.4 (SAS Institute Inc., Cary, NC, EUA) e pelo software R 4.0.3 (Institute for Statistics and Mathematics, Viena, Áustria). P<0,05 foi considerado estatisticamente significativo.

## Resultados

### Características dos participantes

**Figura 1:**
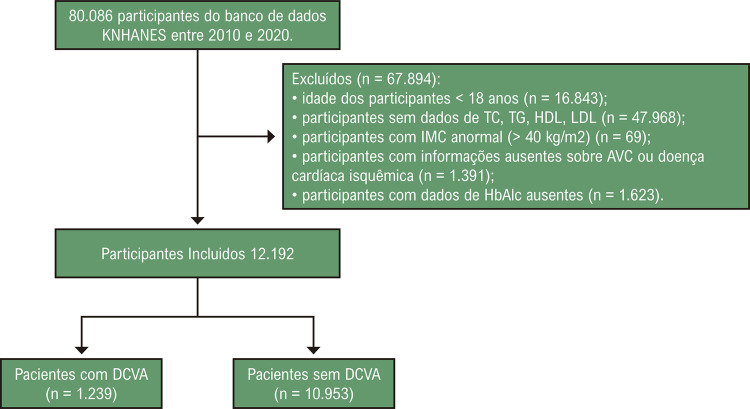
– Fluxograma de sujeitos incluídos. KNHANES, Korea National Health and Nutrition Examination Survey. CT: colesterol total; TG: triglicerídeo; HDL: lipoproteína de alta densidade; LDL: lipoproteína de baixa densidade; IMC: índice de massa corporal; HbA1c: hemoglobina glicada; DCVA: doença cardiovascular aterosclerótica.

Um total de 80.086 participantes foram extraídos do banco de dados KNHANES entre 2010 e 2020. Foram excluídos 72.268 participantes, incluindo 16.843 participantes com menos de 18 anos, 47.968 participantes com dados ausentes de CT, TG, HDL, LDL, 69 participantes com IMC anormal (IMC ≥ 40 kg/m^2^), 1.391 participantes com informações ausentes sobre acidente vascular cerebral ou doença cardíaca isquêmica e 1.623 participantes com dados ausentes de HbA1c (Figura 1). Um total de 12.192 participantes com dados completos foram incluídos neste estudo, dos quais 1.239 (10,16%) tinham DCVA (Tabela 1). As características detalhadas dos participantes são mostradas na Tabela 1. Diferenças estatísticas entre participantes com e sem DCVA foram observadas em idade, sexo, IMC, nível de educação, medicamento hipolipemiante, hipertensão, diabetes, creatinina, HbA1c, não-HDL-C, LDL-C, HDL-C, TG, CT e EsdLDL-C (Tabela 1).

**Tabela 1:** – Características de todos os participantes

Variáveis	Total (N=12192)	Não-DCVA (N=10953)	DCVA (N=1239)	p
Idade, anos, média (±DP)	50,02 (±15,28)	49,87 (±15,57)	51,39 (±12,34)	<0,001
Sexo, n (%)				0,015
Masc.	6504 (53,35)	5802 (52,97)	702 (56,66)	
Fem.	5688 (46,65)	5151 (47,03)	537 (43,34)	
IMC, Média (±DP)	24,64 (±3,47)	24,61 (±3,48)	24,94 (±3,41)	0,001
Renda, n (%)				0,485
Q1	3043 (24,96)	2754 (25,14)	289 (23,33)	
Q2	3084 (25h30)	2767 (25,26)	317 (25,59)	
Q3	3012 (24,70)	2705 (24,70)	307 (24,78)	
Q4	2971 (24,37)	2651 (24.20)	320 (25,83)	
Desconhecido	82 (0,67)	76 (0,69)	6 (0,48)	
Educação, n (%)				<0,001
Seodang/Hanhak	2180 (17,88)	1998 (18.24)	182 (14,69)	
Sem educação	1246 (10.22)	1124 (10.26)	122 (9,85)	
Escola primária	4004 (32,84)	3588 (32,76)	416 (33,58)	
Escola secundária	3965 (32,52)	3508 (32,03)	457 (36,88)	
Desconhecido	797 (6,54)	735 (6,71)	62 (5,00)	
Bebida, n (%)				0,191
Não	1199 (9,83)	1088 (9,93)	111 (8,96)	
Sim	10630 (87,19)	9531 (87,02)	1099 (88,70)	
Desconhecido	363 (2,98)	334 (3,05)	29 (2,34)	
Tabagismo, n (%)				0,088
Não	8819 (72,33)	7945 (72,54)	874 (70,54)	
Sim	2994 (24,56)	2661 (24,29)	333 (26,88)	
Desconhecido	379 (3.11)	347 (3,17)	32 (2,58)	
Medicamentos hipolipemiantes, n (%)				<0,001
Não	8627 (70,76)	7807 (71,28)	820 (66,18)	
Sim	2668 (21,88)	2324 (21,22)	344 (27,76)	
Desconhecido	897 (7,36)	822 (7,50)	75 (6,05)	
Hipertensão, n (%)				<0,001
Não	9267 (76,01)	8620 (78,70)	647 (52,22)	
Sim	2381 (19,53)	1832 (16,73)	549 (44,31)	
Desconhecido	544 (4,46)	501 (4,57)	43 (3,47)	
Diabetes, n (%)				<0,001
Não	10424 (85,50)	9408 (85,89)	1016 (82,00)	
Sim	1148 (9,42)	979 (8,94)	169 (13,64)	
Desconhecido	620 (5,09)	566 (5,17)	54 (4,36)	
Creatinina, mg/dL,M (Q1, Q3)	0,83 (0,70, 0,96)	0,83 (0,70, 0,96)	0,85 (0,72, 0,98)	0,001
BUN, mg/dL,M (Q1, Q3)	14h00 (12h00, 17h00)	14h00 (12h00, 17h00)	14h00 (12h00, 17h00)	0,203
HbA1c, %,M (Q1, Q3)	5,60 (5,40, 6,00)	5,60 (5,30, 6,00)	5,70 (5,40, 6,10)	<0,001
Não-HDL-C, mg/dL, Média (±DP)	150,83 (±39,38)	150,46 (±39,14)	154,05 (±41,26)	0,002
Esb-LDL-C, mg/dL, Média (±DP)	42,43 (±14,75)	42,27 (±14,71)	43,88 (±15,02)	<0,001
LDL-C, mg/dL, Média (± DP)	115,12 (±33,43)	114,91 (±33,32)	116,97 (±34,35)	0,040
TG, mg/dL,M (Q1, Q3)	205,00 (101,00, 267,00)	204,00 (101,00, 267,00)	211,00 (110,50, 272,00)	0,003
CT, mg/dL, Média (± DP)	197,93 (±38,83)	197,66 (±38,56)	200,33 (±41,07)	0,021
HDL-C, mg/dL, Média (±DP)	47,10 (±12,29)	47,19 (±12,35)	46,29 (±11,71)	0,010

DCVA: doença cardiovascular aterosclerótica; IMC: índice de massa corporal; quartis, Q1, Q2, Q3, Q4; BUN: nitrogênio ureico no sangue; HbA1c: hemoglobina glicada; HDL-C: lipoproteína de alta densidade; LDL-C: colesterol de lipoproteína de baixa densidade; TG: triglicerídeo; CT: colesterol total; Esd-LDL-C: lipoproteína de baixa densidade pequena estimada.

### Relação entre EsdLDL-C e risco de DCVA

**Figura 2:**
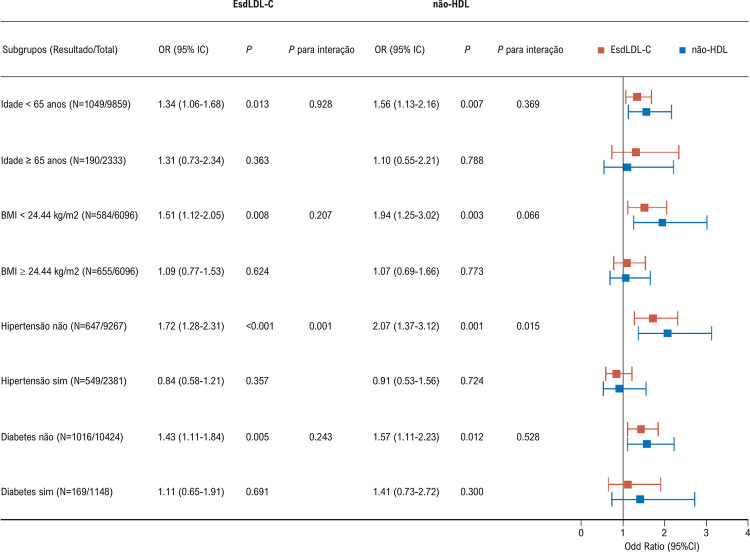
– Relação entre EsdLDL-C e não-HDL e risco de DCVA em diferentes populações. Esd-LDL-C: lipoproteína de baixa densidade pequena estimada; HDL: lipoproteína de alta densidade; DCVA: doença cardiovascular aterosclerótica; IMC: índice de massa corporal.

A Tabela 2 mostra a associação de EsdLDL-C, não-HDL-C, HDL-C, LDL-C, TG e CT com o risco de DCVA, acidente vascular cerebral e doença cardíaca isquêmica. Níveis elevados de EsdLDL-C, não-HDL-C e TG foram associados a um risco aumentado de DCVA, enquanto níveis elevados de HDL-C reduziram o risco de DCVA. Além disso, níveis elevados de EsdLDL-C, não-HDL-C e TG foram relacionados a um risco maior de doença cardíaca isquêmica, mas nenhuma relação foi observada entre EsdLDL-C, não-HDL-C, HDL-C, LDL-C, TG e CT e risco de acidente vascular cerebral. Além disso, o teste DeLong indicou que a capacidade do EsdLDL-C de prever o risco de DCVA foi ligeiramente melhor do que a do CT (AUC: 0,527 vs. 0,515; P=0,039), mas nenhuma diferença significativa foi encontrada quando comparado a outros indicadores. Análises de subgrupos foram realizadas para avaliar a relação entre EsdLDL-C e não-HDL-C e risco de DCVA em diferentes populações com base na idade, IMC, hipertensão e diabetes (Figura 2). Níveis elevados de EsdLDL-C foram relacionados a um risco aumentado de DCVA em participantes com idade <65, com IMC <24,44 kg/m^2^ e sem hipertensão ou diabetes. Da mesma forma, níveis elevados de não-HDL-C aumentaram o risco de DCVA em participantes com idade <65, com IMC <24,44 kg/m^2^ e sem hipertensão ou diabetes. Pode haver uma interação entre EsdLDL-C ou não-HDL-C e hipertensão no risco de DCVA.

**Tabela 2:** – Relação entre lipídios e risco de doença cardiovascular aterosclerótica

Resultados	Variáveis	Modelo 1		Modelo 2	
		OR (IC 95%)	p	OR (IC 95%)	p
DCVA	Não-HDL-C	1,63 (1,23-2,18)	0,001	1,46 (1,08-1,98)	0,014
	Esb-LDL-C	1,43 (1,16-1,77)	0,001	1,33 (1,06-1,66)	0,012
	LDL-C	1,15 (0,89-1,49)	0,284	1,13 (0,88-1,46)	0,348
	TG	1,17 (1,05-1,30)	0,004	1,14 (1,01-1,28)	0,030
	CT	1,62 (1,10-2,39)	0,015	1,47 (0,98-2,19)	0,060
	HDL-C	0,62 (0,47-0,81)	<0,001	0,72 (0,55-0,95)	0,019
AVC	Não-HDL-C	1,49 (1,06-2,10)	0,021	1,23 (0,85-1,78)	0,264
	Esb-LDL-C	1,35 (1,05-1,74)	0,019	1,19 (0,90-1,56)	0,213
	LDL-C	1,11 (0,82-1,51)	0,492	1,09 (0,81-1,47)	0,569
	TG	1,08 (0,97-1,21)	0,160	1,02 (0,90-1,15)	0,733
	CT	1,41 (0,89-2,26)	0,147	1,20 (0,74-1,95)	0,459
	HDL-C	0,59 (0,43-0,82)	0,001	0,77 (0,56-1,07)	0,120
Doença cardíaca isquêmica	Não-HDL-C	1,79 (1,15-2,81)	0,011	1,77 (1,09-2,87)	0,021
	Esb-LDL-C	1,56 (1,14-2,13)	0,005	1,51 (1,08-2,11)	0,017
	LDL-C	1,23 (0,81-1,86)	0,333	1,16 (0,76-1,77)	0,506
	TG	1,26 (1,07-1,49)	0,005	1,25 (1,05-1,49)	0,012
	CT	1,91 (1,04-3,51)	0,038	1,86 (0,99-3,49)	0,054
	HDL-C	0,67 (0,45-1,01)	0,057	0,71 (0,45-1,12)	0,143

OR: razão de chances; IC: intervalo de confiança; DCVA: doença cardiovascular aterosclerótica HDL-C: lipoproteína de alta densidade; LDL-C: colesterol de lipoproteína de baixa densidade; TG: triglicerídeos; CT: colesterol total; Esd-LDL-C: lipoproteína de baixa densidade pequena estimada; Modelo 1, modelo de regressão logística univariável; Modelo 2, modelo de regressão logística multivariável que ajusta para (1) idade, creatinina e HbA1c (análise para DCVA); (2) idade, BUN e HbA1c (análise para acidente vascular cerebral); (3) idade e IMC (análise para doença cardíaca isquêmica).

## Discussão

Neste estudo, usamos dados do banco de dados KNHANES para avaliar a relação entre os níveis estimados de sdLDL-C e o risco de DCVA. Os resultados descobriram que níveis elevados de EsdLDL-C estavam associados a um risco aumentado de DCVA. Análises de subgrupos mostraram que pode haver uma interação entre EsdLDL-C ou não-HDL-C e hipertensão no risco de DCVA. Vários estudos documentaram que o nível elevado de sdLDL-C estava associado ao risco de doença cardiovascular.^18-20^ A aterosclerose causada por sdLDL está relacionada a propriedades bioquímicas e biofísicas específicas das partículas de sdLDL.^9^ O pequeno tamanho do sdLDL permite sua penetração na parede arterial e serve como fonte de armazenamento de colesterol e lipídios. O sdLDL circula por mais tempo do que aquelas partículas grandes de LDL que são eliminadas da corrente sanguínea pela interação com receptores de LDL, o que aumenta o potencial aterogênico do sdLDL no plasma.^21^ Um estudo recente indicou que o nível de sdLDL-C foi um biomarcador melhor para a avaliação de doença cardíaca coronária do que o nível de LDL-C.^22^ A medição do sdLDL-C recebeu atenção devido ao seu papel na previsão de DCVA. Os métodos tradicionais de medição do sdLDL-C dependem de testes laboratoriais adicionais, como ultracentrifugação ou eletroforese em gel de gradiente, que são específicos do equipamento ou demorados.^14^ Ito et al. desenvolveram uma nova técnica de detecção laboratorial para níveis de sdLDL-C, que usa um analisador automático para detecção e economiza tempo de detecção.^23^ Alguns estudos propuseram usar indicadores bioquímicos relacionados ao sdLDL-C, como LDL-C e TG, para desenvolver uma fórmula para estimar o sdLDL-C para reduzir testes laboratoriais adicionais.^15,24^ Sampson et al. forneceram uma nova fórmula para estimar os níveis séricos de sdLDL-C.^15^ Sua fórmula de estimativa de sdLDL-C usou níveis de LDL-C e TG para calcular os níveis de sdLDL-C e não exigiu nenhum teste laboratorial adicional além do painel lipídico padrão. Os dois termos principais na fórmula são (1,43× LDL-C ) e (0,14×(ln⁡(TG)×LDL−C)) . O termo ×(1,43 × LDL-C) ilustra que indivíduos com altos níveis de LDL-C podem ter mais lbLDL. O termo (0,14 × (ln (TG) × LDL-C)) é o termo de interação entre LDL-C e TG, que representa uma maior proporção de colesterol entre sdLDL e lbLDL com o aumento de TG.^15^ O estudo atual validou a aplicabilidade de sua fórmula usando dados de outras populações. Nossos resultados mostraram que os valores de sdLDL-C calculados pela fórmula foram associados ao risco de DCVA na população coreana. As análises de subgrupos descobriram que a relação entre o aumento do nível de sdLDL-C e o risco de DCVA foi observada em participantes com idade <65 anos, com IMC <24,44 kg/m^2^ e sem hipertensão ou diabetes. No entanto, apenas uma interação entre EsdLDL-C e hipertensão no risco de DCVA foi encontrada, sugerindo que os resultados sobre o risco de sdLDL-C e DCVA em subgrupos de idade, IMC e diabetes precisam ser interpretados com cautela. A associação entre Esd-LDL e risco de DCVA em nosso estudo foi consistente com estudos anteriores.^25,26^ Também analisamos a associação de não-HDL-C, HDL-C, LDL-C, TG e CT com o risco de DCVA, acidente vascular cerebral e doença cardíaca isquêmica. Além disso, nossos resultados descobriram que a capacidade do EsdLDL-C de prever o risco de DCVA foi ligeiramente melhor do que a do não-HDL-C, HDL-C, LDL-C, TG e CT. Estudos anteriores também sugeriram que o sdLDL-C estava mais fortemente associado ao risco de DCVA do que o LDL-C.^9,22,27^ Esses resultados sugerem que o sdLDL-C estimado está similarmente associado ao risco de DCVA. Para testes laboratoriais complexos de níveis de sdLDL-C, o sdLDL-C estimado pode ser uma alternativa aos testes laboratoriais de sdLDL-C para avaliação de risco de DCVA, o que não apenas evita os testes complexos de sdLDL-C, mas também permite a estimativa rápida dos níveis de sdLDL-C com base no painel lipídico padrão. Uma nova fórmula para estimar o sdLDL-C foi validada com base nos dados do banco de dados KNHANES. Analisamos a associação entre sdLDL-C e LDL-C e risco de DCVA. Em seguida, a relação entre sdLDL-C e LDL-C e risco de DCVA foi analisada posteriormente com base na idade, IMC, hipertensão e diabetes. No entanto, algumas limitações deste estudo devem ser consideradas. Primeiro, a fórmula foi baseada em indivíduos em jejum, e sua precisão em indivíduos não em jejum também deve ser verificada. Segundo, não podemos comparar a diferença entre o valor calculado de sdLDL-C usando a fórmula e o valor real de sdLDL-C devido à falta de dados de sdLDL-C no banco de dados KNHANES. Terceiro, embora tenhamos considerado a interferência de muitos fatores de confusão, ainda havia alguns fatores de confusão que podem afetar os resultados que não foram considerados, como hábitos alimentares, níveis de atividade física ou histórico familiar de DCV. Quarto, este estudo foi um estudo transversal, e não foi possível analisar DCVA com base na duração da exposição do paciente ao sdLDL-C.

## Conclusões

Uma fórmula proposta recentemente para estimar o sdLDL-C foi validada com base em outras populações. Os resultados indicaram que níveis elevados de EsdLDL-C estavam associados a um risco aumentado de DCVA. Análises de subgrupos descobriram que níveis elevados de sdLDL-C estavam relacionados a um risco aumentado de DCVA em participantes com idade <65 anos, com IMC <24,44 kg/m^2^ e sem hipertensão ou diabetes. O sdLDL-C estimado pode ser uma alternativa à medição do sdLDL-C para avaliação do risco de DCVA.

## References

[1] Libby P, Buring JE, Badimon L, Hansson GK, Deanfield J, Bittencourt MS (2019). Atherosclerosis. Nat Rev Dis Primers.

[2] Roth GA, Mensah GA, Johnson CO, Addolorato G, Ammirati E, Baddour LM (2020). Global Burden of Cardiovascular Diseases and Risk Factors, 1990-2019: Update from the GBD 2019 Study. J Am Coll Cardiol.

[3] World Health Organization (2024). Cardiovascular diseases.

[4] Ference BA, Ginsberg HN, Graham I, Ray KK, Packard CJ, Bruckert E (2017). Low-density Lipoproteins Cause Atherosclerotic Cardiovascular Disease. 1. Evidence from Genetic, Epidemiologic, and Clinical Studies. A Consensus Statement from the European Atherosclerosis Society Consensus Panel. Eur Heart J.

[5] Catapano AL, Graham I, De Backer G, Wiklund O, Chapman MJ, Drexel H (2016). 2016 ESC/EAS Guidelines for the Management of Dyslipidaemias. Eur Heart J.

[6] Sandesara PB, Virani SS, Fazio S, Shapiro MD (2019). The Forgotten Lipids: Triglycerides, Remnant Cholesterol, and Atherosclerotic Cardiovascular Disease Risk. Endocr Rev.

[7] Holmes MV, Asselbergs FW, Palmer TM, Drenos F, Lanktree MB, Nelson CP (2015). Mendelian Randomization of Blood Lipids for Coronary Heart Disease. Eur Heart J.

[8] Di Angelantonio E, Gao P, Pennells L, Kaptoge S, Caslake M, Thompson A (2012). Lipid-related Markers and Cardiovascular Disease Prediction. JAMA.

[9] Ivanova EA, Myasoedova VA, Melnichenko AA, Grechko AV, Orekhov AN (2017). Small Dense Low-density Lipoprotein as Biomarker for Atherosclerotic Diseases. Oxid Med Cell Longev.

[10] Gerber PA, Thalhammer C, Schmied C, Spring S, Amann-Vesti B, Spinas GA (2013). Small, Dense LDL Particles Predict Changes in Intima Media Thickness and Insulin Resistance in Men with Type 2 Diabetes and Prediabetes--A Prospective Cohort Study. PLoS One.

[11] Vekic J, Zeljkovic A, Stefanovic A, Jelic-Ivanovic Z, Spasojevic-Kalimanovska V (2019). Obesity and Dyslipidemia. Metabolism.

[12] Goldberg R, Temprosa M, Otvos J, Brunzell J, Marcovina S, Mather K (2013). Lifestyle and Metformin Treatment Favorably Influence Lipoprotein Subfraction Distribution in the Diabetes Prevention Program. J Clin Endocrinol Metab.

[13] Higashioka M, Sakata S, Honda T, Hata J, Shibata M, Yoshida D (2021). The Association of Small Dense Low-density Lipoprotein Cholesterol and Coronary Heart Disease in Subjects at High Cardiovascular Risk. J Atheroscler Thromb.

[14] Hirayama S, Miida T (2012). Small Dense LDL: An Emerging Risk Factor for Cardiovascular Disease. Clin Chim Acta.

[15] Sampson M, Wolska A, Warnick R, Lucero D, Remaley AT (2021). A New Equation Based on the Standard Lipid Panel for Calculating Small Dense Low-density Lipoprotein-Cholesterol and Its Use as a Risk-enhancer Test. Clin Chem.

[16] Oh K, Kim Y, Kweon S, Kim S, Yun S, Park S (2021). Korea National Health and Nutrition Examination Survey, 20th Anniversary: Accomplishments and future Directions. Epidemiol Health.

[17] Kweon S, Kim Y, Jang MJ, Kim Y, Kim K, Choi S (2014). Data Resource Profile: the Korea National Health and Nutrition Examination Survey (KNHANES). Int J Epidemiol.

[18] Krauss RM (2010). Lipoprotein Subfractions and Cardiovascular Disease Risk. Curr Opin Lipidol.

[19] Rizzo M, Berneis K (2006). The Clinical Relevance of Low-density-lipoproteins Size Modulation by Statins. Cardiovasc Drugs Ther.

[20] Arai H, Kokubo Y, Watanabe M, Sawamura T, Ito Y, Minagawa A (2013). Small Dense Low-density Lipoproteins Cholesterol Can Predict Incident Cardiovascular Disease in an Urban Japanese Cohort: The Suita Study. J Atheroscler Thromb.

[21] Packard C, Caslake M, Shepherd J (2000). The Role of Small, Dense Low Density Lipoprotein (LDL): A New Look. Int J Cardiol.

[22] Higashioka M, Sakata S, Honda T, Hata J, Yoshida D, Hirakawa Y (2020). Small Dense Low-density Lipoprotein Cholesterol and the Risk of Coronary Heart Disease in a Japanese Community. J Atheroscler Thromb.

[23] Ito Y, Fujimura M, Ohta M, Hirano T (2011). Development of a Homogeneous Assay for Measurement of Small Dense LDL Cholesterol. Clin Chem.

[24] Srisawasdi P, Chaloeysup S, Teerajetgul Y, Pocathikorn A, Sukasem C, Vanavanan S (2011). Estimation of plasma Small Dense LDL Cholesterol from Classic Lipid Measures. Am J Clin Pathol.

[25] Duran EK, Aday AW, Cook NR, Buring JE, Ridker PM, Pradhan AD (2020). Triglyceride-Rich Lipoprotein Cholesterol, Small Dense LDL Cholesterol, and Incident Cardiovascular Disease. J Am Coll Cardiol.

[26] Zhou P, Liu J, Wang L, Feng W, Cao Z, Wang P (2020). Association of Small Dense Low-density Lipoprotein Cholesterol with Stroke Risk, Severity and Prognosis. J Atheroscler Thromb.

[27] Tsai MY, Steffen BT, Guan W, McClelland RL, Warnick R, McConnell J (2014). New Automated Assay of Small Dense Low-density Lipoprotein Cholesterol Identifies Risk of Coronary Heart Disease: The Multi-ethnic Study of Atherosclerosis. Arterioscler Thromb Vasc Biol.

