# Supplementary material for: Associação entre Estimativa de Colesterol de Lipoproteína de Baixa Densidade (sdLDL-C) e Risco de Doença Cardiovascular Aterosclerótica
Source: Arq Bras Cardiol. 2025 Jan 22;122(1):e20240265. [Article in Portuguese] doi: 10.36660/abc.20240265 (PMC11805573; doi:10.36660/abc.20240265)
Supplement: Translation

# Translation: Association between Estimated Small Dense Low-Density Lipoprotein-cholesterol (sdLDL-C) and Atherosclerotic Cardiovascular Disease Risk

**DOI:** 10.36660/abc.20240265i

**Authors:** Shutang Zhang, Jinjie Du, Peng Wang, Min Lei, Canye Zhong, Yang Ou, Zhen Sun

**Affiliations:** 1 China Chongqing University Fuling Hospital, Chongqing Clinical Research Center for Geriatric Diseases – Geriatrics, Chongqing – China

**Keywords:** Lipoproteins, Atherosclerosis, Risk Factors

## Abstract

**Background:**

A new formula for estimating small, dense, low-density lipoprotein cholesterol (sdLDL-C) based on the results of the standard lipid panel is proposed.

**Objectives:**

To assess the association between estimated sdLDL-C (EsdLDL-C) and atherosclerotic cardiovascular disease (ASCVD) risk.

**Methods:**

A total of 12,192 participants from the Korea National Health and Nutrition Examination Survey (KNHANES) database between 2010 and 2020 were included in this cross-sectional study. EsdLDL-C was calculated as EsdLDL-C= LDL-C - [1.43 × LDL-C - (0.14 × (ln (TG) × LDL-C)) - 8.99]. Logistic regression analyses were utilized to assess the association between EsdLDL-C and ASCVD risk. Subgroup analyses were performed based on age, body mass index (BMI), hypertension, and diabetes. An odds ratio (OR) with a 95% confidence interval (CI) was used for evaluation. P<0.05 was considered statistically significant.

**Results:**

Among 12,192 participants, 1,239 (10.16%) had ASCVD. The mean sdLDL-C of participants was estimated to be 42.43±14.75 mg/dL using the formula. Elevated EsdLDL-C levels (OR=1.33; 95%CI, 1.06-1.66) were associated with an increased risk of ASCVD. Subgroup analyses found that there may be an interaction between EsdLDL-C (P_interaction_= 0.001) or non-HDL-C (P_interaction_= 0.015) and hypertension on ASCVD risk.

**Conclusions:**

Elevated estimated sdLDL-C levels were associated with the risk of ASCVD, and estimated sdLDL-C might be an alternative to sdLDL-C measurement for ASCVD risk assessment.

## Introduction

Atherosclerotic cardiovascular disease (ASCVD) is an insidious, chronic disease that usually processes to an advanced stage when symptoms appear.^1^ The most frequent diseases of ASCVD are coronary heart disease and stroke, which are the leading causes of death.^2^ The World Health Organization reported that ASCVD has become the leading cause of death globally, claiming approximately 17.9 million lives each year.^1^ Identifying and monitoring biomarkers associated with ASCVD plays an important role in its primary and secondary prevention.

Increased low-density lipoprotein cholesterol (LDL-C) is a key causal factor in the development and progression of ASCVD.^3,4^ Previous studies have demonstrated that individuals with low LDL-C levels have a lower incidence of ASCVD than those with high LDL-C levels.^5-7^ LDL is composed of several subclasses of particles with different sizes and densities, including large buoyant (lb) and intermediate and small dense (sd) LDLs.^8^ However, sdLDL may be a better biomarker than other subtypes for ASCVD risk in different LDL subtypes.^9,10^ sdLDL was reported to be associated with a variety of diseases, including metabolic disorders, obesity, and type 2 diabetes, and is considered a risk factor for coronary heart disease.^11-13^ Therefore, the measurement of sdLDL-C levels is of great significance in monitoring ASCVD risk. Traditional methods of measuring sdLDL-C relied on complex ultracentrifugation or gradient gel electrophoresis.^14^ The special equipment required for measurement and long assay time limited the clinical application of sdLDL measurement. Sampson et al. developed a new equation for estimating sdLDL-C based on the results of the standard lipid panel with a determination coefficient of 0.745.^15^ However, their formula was only established in the American population, and the adaptation and estimated effect in other populations remains unclear.

Herein, we hypothesized that Sampson et al.’s formula for estimating sdLDL was also applicable to other populations and was associated with ASCVD risk. Data from the Korea National Health and Nutrition Examination Survey (KNHANES) database were used to assess the association between sdLDL and ASCVD risk.

## Methods

### Data acquisition and participants

Data used in the cross-sectional study were extracted from the KNHANES database between 2010 and 2020.^16^ KNHANES database is a national surveillance system to assess the health and nutritional status of Koreans by collecting information on socioeconomic status, health-related behaviors, quality of life, healthcare utilization, anthropometric measurements, and biochemical and clinical profiles of non-communicable diseases.^17^ KNHANES is a nationally representative cross-sectional survey conducted annually, each survey year including a new sample of approximately 10,000 people aged 1 year and older. The survey consists of three parts: health interview, health examination, and nutrition survey. The health interview and health examination are conducted in a mobile examination center by trained medical staff and interviewers. One week after the health examination, dietitians went to the participants’ homes for a nutritional survey. Participants aged more than 18 and with complete cholesterol information were included. Participants were excluded based on the following criteria: (1) with abnormal BMI values (BMI >40kg/m^2^); (2) with missing information on glycated hemoglobin (HbA1c); (3) with missing information on stroke or ischemic heart disease. Protocols of KNHANES were approved by the Korea Centers for Disease Control and Prevention (KCDC). All data used in this study are anonymized in the KNHANES database and did not involve human interventions. Therefore, this study did not require additional Institutional Review Board approval.

### Data collection

Demographic and biochemical indicators of participants include age (≥18 years), gender (male and female), body mass index (BMI), income level [quartiles (Q1, Q2, Q3, Q4), and unknown], education level (seodang/hanhak, uneducated, elementary school, middle school, and unknown), drinking alcohol (yes, no, and unknown), smoking (yes and no), lipid-lowering drug (yes, no, and unknown), hypertension (yes, no, and unknown), diabetes (yes, no, and unknown), creatinine, blood urea nitrogen (BUN), HbA1c, LDL-C, high-density lipoprotein (HDL-C), non-HDL-C, triglyceride (TG), total cholesterol (TC), and estimated sdLDL-C (EsdLDL-C) were collected. Non-HDL-C was calculated as non-HDL-C = TC - HDL-C. All lipid levels were measured by direct blood sampling by a nurse in the context of participants having eaten dinner the previous day.

### Definition and measurement

### ASCVD

ASCVD events include myocardial infarction, angina, percutaneous coronary intervention, coronary artery bypass graft, congestive heart failure, peripheral vascular disease, stroke, and transient ischemic attack. Due to limitations of the KNHANES database, ASCVD events included ischemic heart disease, myocardial infarction, angina pectoris, and stroke. In the KNHANES database, ischemic heart disease was determined by the question, “Have you ever been diagnosed with myocardial infarction or angina by your doctor?”. Therefore, ASCVD events in this study included only ischemic heart disease and stroke.

### sdLDL-C

sdLDL-C was calculated from Sampson et al.^15^. The relevant formulas were as follows:


$$\begin{array}{cc} & \;\text{ lbLDL-C }=1.43\times \text{ LDL-C }-(0.14\times (\ln(TG)\times \text{ LDL-C }))\\ & -8.99 \end{array}$$ (1)

sdLDL-C = LDL-C − IbLDL-C (2)

### Statistical analysis

Continuous variables were tested for normality using the skewness and kurtosis method, and the Levene test was used to test the homogeneity of variance. Normally distributed continuous variables were described by mean and standard deviation (SD). Comparison between groups of continuous variables with homogeneous variances was performed using unpaired Student’s t-test, and continuous variables with heteroscedasticity were performed using Satterthwaite t-test. Non-normally distributed continuous variables were described by median and quartile [M (Q1, Q3)], and the Wilcoxon rank sum test was used for inter-group comparisons. Categorical variables were presented by numbers and the constituent ratio [n (%)], and the comparison between groups was performed using the Chi-square test or Fisher exact test.

A difference analysis between the characteristics of participants with and without ASCVD was performed. Variables with p < 0.05 in the difference analysis were screened by bidirectional stepwise regression, and the final screened variables were adjusted in multivariable logistic regression analysis. Univariable and multivariable logistic regression analyses were used to assess the association of EsdLDL-C, non-HDL-C, HDL-C, LDL-C, TG, and TC with the risk of ASCVD, stroke, and ischemic heart disease. The associations were expressed as odds ratio (OR) with 95% confidence interval (CI). The area under the receiver operating characteristic curve (AUC) was used to evaluate the ability of EsdLDL-C, non-HDL-C, HDL-C, LDL-C, TG, and TC to predict the risk of ASCVD, and the DeLong test was used to compare the differences in AUC between these indicators. Subgroup analysis was conducted based on age (<65 and ≥65), BMI (<24.44 and ≥24.44 kg/m^2^), hypertension (no and yes), and diabetes (no and yes). Statistical analyses were performed by SAS 9.4 software (SAS Institute Inc., Cary, NC, USA) and R 4.0.3 software (Institute for Statistics and Mathematics, Vienna, Austria). P<0.05 was considered statistically significant.

## Results

### Characteristics of participants

A total of 80,086 participants were extracted from the KNHANES database between 2010 and 2020. There were 72,268 participants excluded, including 16,843 participants less than 18 years, 47,968 participants with missing TC, TG, HDL, LDL data, 69 participants with abnormal BMI (BMI ≥ 40 kg/m^2^), 1,391 participants with missing information on stroke or ischemic heart disease, and 1,623 participants with missing HbA1c data (Figure 1). A total of 12,192 participants with complete data were included in this study, of whom 1,239 (10.16%) had ASCVD (Table 1). The detailed characteristics of participants are shown in Table 1.

**Figure 1:** – Flowchart of included subjects. KNHANES, the Korea National Health and Nutrition Examination Survey. TC: total cholesterol; TG: triglyceride; HDL: high-density lipoprotein; LDL: low-density lipoprotein; BMI: body mass index; HbA1c: glycated hemoglobin; ASCVD: atherosclerotic cardiovascular disease.

**Table 1:** – Characteristics of all participants

Variables	Total (N=12192)	Non-ASCVD (N=10953)	ASCVD (N=1239)	p
Age, years, Mean (± SD)	50.02 (±15.28)	49.87 (±15.57)	51.39 (±12.34)	<0.001
Sex, n (%)				0.015
Male	6504 (53.35)	5802 (52.97)	702 (56.66)	
Female	5688 (46.65)	5151 (47.03)	537 (43.34)	
BMI, Mean (± SD)	24.64 (±3.47)	24.61 (±3.48)	24.94 (±3.41)	0.001
Income, n (%)				0.485
Q1	3043 (24.96)	2754 (25.14)	289 (23.33)	
Q2	3084 (25.30)	2767 (25.26)	317 (25.59)	
Q3	3012 (24.70)	2705 (24.70)	307 (24.78)	
Q4	2971 (24.37)	2651 (24.20)	320 (25.83)	
Unknown	82 (0.67)	76 (0.69)	6 (0.48)	
Education, n (%)				<0.001
Seodang/hanhak	2180 (17.88)	1998 (18.24)	182 (14.69)	
Uneducated	1246 (10.22)	1124 (10.26)	122 (9.85)	
Elementary school	4004 (32.84)	3588 (32.76)	416 (33.58)	
Middle school	3965 (32.52)	3508 (32.03)	457 (36.88)	
Unknown	797 (6.54)	735 (6.71)	62 (5.00)	
Drinking, n (%)				0.191
No	1199 (9.83)	1088 (9.93)	111 (8.96)	
Yes	10630 (87.19)	9531 (87.02)	1099 (88.70)	
Unknown	363 (2.98)	334 (3.05)	29 (2.34)	
Smoking, n (%)				0.088
No	8819 (72.33)	7945 (72.54)	874 (70.54)	
Yes	2994 (24.56)	2661 (24.29)	333 (26.88)	
Unknown	379 (3.11)	347 (3.17)	32 (2.58)	
Lipid-lowering drugs, n (%)				<0.001
No	8627 (70.76)	7807 (71.28)	820 (66.18)	
Yes	2668 (21.88)	2324 (21.22)	344 (27.76)	
Unknown	897 (7.36)	822 (7.50)	75 (6.05)	
Hypertension, n (%)				<0.001
No	9267 (76.01)	8620 (78.70)	647 (52.22)	
Yes	2381 (19.53)	1832 (16.73)	549 (44.31)	
Unknown	544 (4.46)	501 (4.57)	43 (3.47)	
Diabetes, n (%)				<0.001
No	10424 (85.50)	9408 (85.89)	1016 (82.00)	
Yes	1148 (9.42)	979 (8.94)	169 (13.64)	
Unknown	620 (5.09)	566 (5.17)	54 (4.36)	
Creatinine, mg/dL, M (Q1, Q3)	0.83 (0.70, 0.96)	0.83 (0.70, 0.96)	0.85 (0.72, 0.98)	0.001
BUN, mg/dL, M (Q1, Q3)	14.00 (12.00, 17.00)	14.00 (12.00, 17.00)	14.00 (12.00, 17.00)	0.203
HbA1c, %, M (Q1, Q3)	5.60 (5.40, 6.00)	5.60 (5.30, 6.00)	5.70 (5.40, 6.10)	<0.001
Non-HDL-C, mg/dL, Mean (± SD)	150.83 (±39.38)	150.46 (±39.14)	154.05 (±41.26)	0.002
Esb-LDL-C, mg/dL, Mean (± SD)	42.43 (±14.75)	42.27 (±14.71)	43.88 (±15.02)	<0.001
LDL-C, mg/dL, Mean (± SD)	115.12 (±33.43)	114.91 (±33.32)	116.97 (±34.35)	0.040
TG, mg/dL, M (Q1, Q3)	205.00 (101.00, 267.00)	204.00 (101.00, 267.00)	211.00 (110.50, 272.00)	0.003
TC, mg/dL, Mean (± SD)	197.93 (±38.83)	197.66 (±38.56)	200.33 (±41.07)	0.021
HDL-C, mg/dL, Mean (± SD)	47.10 (±12.29)	47.19 (±12.35)	46.29 (±11.71)	0.010

ASCVD: atherosclerotic cardiovascular disease; BMI: body mass index; quartiles, Q1, Q2, Q3, Q4; BUN: blood urea nitrogen; HbA1c: glycated hemoglobin; HDL-C: high-density lipoprotein; LDL-C: low-density lipoprotein cholesterol; TG: triglyceride; TC: total cholesterol; Esd-LDL-C: estimated small dense low-density lipoprotein.

Statistical differences between participants with and without ASCVD were observed in age, sex, BMI, education level, lipid-lowering drug, hypertension, diabetes, creatinine, HbA1c, non-HDL-C, LDL-C, HDL-C, TG, TC, and EsdLDL-C (Table 1).

### Relationship between EsdLDL-C and ASCVD risk

Table 2 shows the association of EsdLDL-C, non-HDL-C, HDL-C, LDL-C, TG, and TC with the risk of ASCVD, stroke, and ischemic heart disease. Elevated EsdLDL-C, non-HDL-C, and TG levels were associated with an increased ASCVD risk, whereas elevated HDL-C levels reduced the risk of ASCVD. In addition, elevated EsdLDL-C, non-HDL-C, and TG levels were related to a higher risk of ischemic heart disease, but no relationship was observed between EsdLDL-C, non-HDL-C, HDL-C, LDL-C, TG, and TC and stroke risk. In addition, the DeLong test indicated that the ability of EsdLDL-C to predict ASCVD risk was slightly better than that of TC (AUC: 0.527 vs. 0.515; p=0.039), but no significant differences were found when compared to other indicators.

**Table 2:** – Relationship between lipids and atherosclerotic cardiovascular disease (ASCVD) risk

Outcomes	Variables	Model 1		Model 2	
		OR (95% CI)	p	OR (95% CI)	p
ASCVD	Non-HDL-C	1.63 (1.23-2.18)	0.001	1.46 (1.08-1.98)	0.014
	Esb-LDL-C	1.43 (1.16-1.77)	0.001	1.33 (1.06-1.66)	0.012
	LDL-C	1.15 (0.89-1.49)	0.284	1.13 (0.88-1.46)	0.348
	TG	1.17 (1.05-1.30)	0.004	1.14 (1.01-1.28)	0.030
	TC	1.62 (1.10-2.39)	0.015	1.47 (0.98-2.19)	0.060
	HDL-C	0.62 (0.47-0.81)	<0.001	0.72 (0.55-0.95)	0.019
Stroke	Non-HDL-C	1.49 (1.06-2.10)	0.021	1.23 (0.85-1.78)	0.264
	Esb-LDL-C	1.35 (1.05-1.74)	0.019	1.19 (0.90-1.56)	0.213
	LDL-C	1.11 (0.82-1.51)	0.492	1.09 (0.81-1.47)	0.569
	TG	1.08 (0.97-1.21)	0.160	1.02 (0.90-1.15)	0.733
	TC	1.41 (0.89-2.26)	0.147	1.20 (0.74-1.95)	0.459
	HDL-C	0.59 (0.43-0.82)	0.001	0.77 (0.56-1.07)	0.120
Ischemic heart disease	Non-HDL-C	1.79 (1.15-2.81)	0.011	1.77 (1.09-2.87)	0.021
	Esb-LDL-C	1.56 (1.14-2.13)	0.005	1.51 (1.08-2.11)	0.017
	LDL-C	1.23 (0.81-1.86)	0.333	1.16 (0.76-1.77)	0.506
	TG	1.26 (1.07-1.49)	0.005	1.25 (1.05-1.49)	0.012
	TC	1.91 (1.04-3.51)	0.038	1.86 (0.99-3.49)	0.054
	HDL-C	0.67 (0.45-1.01)	0.057	0.71 (0.45-1.12)	0.143

OR: odds ratio; CI: confidence interval; HDL-C: high-density lipoprotein; LDL-C: low-density lipoprotein cholesterol; TG: triglyceride; TC: total cholesterol; Esd-LDL-C: estimated small dense low-density lipoprotein; Model 1, univariable logistic regression model; Model 2, multivariable logistic regression model that adjusts for (1) age, creatinine, and HbA1c (analysis for ASCVD); (2) age, BUN, and HbA1c (analysis for stroke); (3) age and BMI (analysis for ischemic heart disease).

Subgroup analyses were performed to assess the relationship between EsdLDL-C and non-HDL-C and ASCVD risk in different populations based on age, BMI, hypertension, and diabetes (Figure 2). Elevated EsdLDL-C levels were related to an increased risk of ASCVD in participants aged <65, with a BMI <24.44 kg/m^2^, and without hypertension or diabetes. Similarly, elevated non-HDL-C levels increased the risk of ASCVD in participants aged <65, with a BMI <24.44 kg/m^2^, and without hypertension or diabetes. There may be an interaction between EsdLDL-C or non-HDL-C and hypertension on ASCVD risk.

**Figure 2:** – Relationship between EsdLDL-C and non-HDL and ASCVD risk in different populations. Esd-LDL-C: estimated small dense low-density lipoprotein; HDL: high-density lipoprotein; ASCVD: atherosclerotic cardiovascular disease; BMI: body mass index.

## Discussion

In this study, we used data from the KNHANES database to assess the relationship between estimated sdLDL-C levels and the risk of ASCVD. The results found that elevated EsdLDL-C levels were associated with an increased ASCVD risk. Subgroup analyses showed that there may be an interaction between EsdLDL-C or non-HDL-C and hypertension on ASCVD risk.

Several studies have documented that elevated sdLDL-C level was associated with cardiovascular disease risk.^18-20^ Atherosclerosis caused by sdLDL is related to specific biochemical and biophysical properties of sdLDL particles.^9^ The small size of sdLDL allows their penetration into the arterial wall and serves as a source of cholesterol and lipid storage. sdLDL circulates longer than those large LDL particles that are cleared from the bloodstream by interacting with LDL receptors, which increases the atherogenic potential of sdLDL in plasma.^21^ A recent study indicated that sdLDL-C level was a better biomarker for the assessment of coronary heart disease than LDL-C level.^22^ The measurement of sdLDL-C has received attention due to its role in predicting ASCVD. Traditional methods of measuring sdLDL-C rely on additional laboratory testing, such as ultracentrifugation or gradient gel electrophoresis, which are equipment-specific or time-consuming.^14^ Ito et al. developed a new laboratory detection technique for sdLDL-C levels, which uses an automatic analyzer for detection and saves detection time.^23^ Some studies proposed to use sdLDL-C-related biochemical indicators such as LDL-C and TG to develop a formula to estimate sdLDL-C to reduce additional laboratory testing.^15,24^

Sampson et al. provided a new formula to estimate serum sdLDL-C levels.^15^ Their sdLDL-C estimating formula used LDL-C and TG levels to calculate sdLDL-C levels and did not require any additional laboratory testing beyond the standard lipid panel. The two main terms in the formula are (1.43× LDL-C ) and (0.14×(ln⁡(TG)× LDL-C )) . The term (1.43× LDL-C ) illustrates that individuals with high LDL-C levels may have more lbLDL. The term (0.14×(ln(TG)× LDL-C) ) is the interaction term between LDL-C and TG, which represents a higher cholesterol ratio between sdLDL and lbLDL with the increase of TG.^15^ The current study validated the applicability of their formula by using data from other populations. Our results showed that sdLDL-C values calculated by the formula were associated with the ASCVD risk in the Korean population. Subgroup analyses found that the relationship between increased sdLDL-C level and ASCVD risk was observed in participants aged <65 years, with a BMI <24.44 kg/m^2^, and without hypertension or diabetes. However, only an interaction between EsdLDL-C and hypertension on ASCVD risk was found, suggesting that the results regarding sdLDL-C and ASCVD risk in age, BMI, and diabetes subgroups need to be interpreted with caution. The association between Esd-LDL and ASCVD risk in our study was consistent with previous studies.^25,26^ We also analyzed the association of non-HDL-C, HDL-C, LDL-C, TG, and TC with the risk of ASCVD, stroke, and ischemic heart disease. In addition, our results found that the ability of EsdLDL-C to predict ASCVD risk was slightly better than that of non-HDL-C, HDL-C, LDL-C, TG, and TC. Previous studies also suggested that sdLDL-C was more strongly associated with ASCVD risk than LDL-C.^9,22,27^ These results suggest that estimated sdLDL-C is similarly associated with ASCVD risk. For complex laboratory testing of sdLDL-C levels, estimated sdLDL-C may be an alternative to laboratory testing of sdLDL-C for ASCVD risk assessment, which not only avoids the complex tests of sdLDL-C but also allows for the rapid estimation of sdLDL-C levels based on the standard lipid panel.

A new formula for estimating sdLDL-C was validated based on the KNHANES database data. We analyzed the association between sdLDL-C and LDL-C and ASCVD risk. Then, the relationship between sdLDL-C and LDL-C and ASCVD risk was further analyzed based on age, BMI, hypertension, and diabetes. However, some limitations of this study should be considered. First, the formula was based on fasted individuals, and its accuracy in non-fasted individuals should also be verified. Second, we cannot compare the difference between the calculated sdLDL-C value using the formula and the true sdLDL-C value due to the lack of sdLDL-C data in the KNHANES database. Third, although we considered the interference of many confounders, there were still some confounders that may affect the results that were not regarded, such as dietary habits, physical activity levels, or family history of CVD. Fourth, this study was a cross-sectional study, and it was not possible to analyze ASCVD based on the duration of patient exposure to sdLDL-C.

## Conclusions

A recently proposed formula for estimating sdLDL-C was validated based on other populations. The results indicated that elevated EsdLDL-C levels were associated with an increased ASCVD risk. Subgroup analyses found that elevated sdLDL-C levels were related to an increased risk of ASCVD in participants aged <65 years, with a BMI <24.44 kg/m^2^, and without hypertension or diabetes. Estimated sdLDL-C might be an alternative to sdLDL-C measurement for ASCVD risk assessment.
